# Practical use of DAT SPECT imaging in diagnosing dementia with Lewy bodies: a US perspective of current guidelines and future directions

**DOI:** 10.3389/fneur.2024.1395413

**Published:** 2024-04-22

**Authors:** Deirdre M. O’Shea, Alexander Arkhipenko, Douglas Galasko, Jennifer G. Goldman, Zulfiqar Haider Sheikh, George Petrides, Jon B. Toledo, James E. Galvin

**Affiliations:** ^1^Department of Neurology, Comprehensive Center for Brain Health, University of Miami, Miller School of Medicine, Coral Gables, FL, United States; ^2^GE HealthCare, Pharmaceutical Diagnostics, Marlborough, MA, United States; ^3^Department of Neurosciences, UC San Diego, San Diego, CA, United States; ^4^JPG Enterprises LLC, Chicago, IL, United States; ^5^Barrow Neurological Institute, Phoenix, AZ, United States; ^6^GE HealthCare, Pharmaceutical Diagnostics, Buckinghamshire, United Kingdom; ^7^Newcastle upon Tyne Hospitals NHS Foundation Trust, Newcastle upon Tyne, United Kingdom; ^8^Nantz National Alzheimer Center, Stanley Appel Department of Neurology, Houston Methodist Hospital, Houston, TX, United States

**Keywords:** dementia with Lewy bodies, Alzheimer’s disease, Parkinson’s disease, ^123^I-ioflupane, DaTscan, SPECT imaging, clinical diagnosis, DLB

## Abstract

**Background:**

Diagnosing Dementia with Lewy Bodies (DLB) remains a challenge in clinical practice. The use of ^123^I-ioflupane (DaTscan^™^) SPECT imaging, which detects reduced dopamine transporter (DAT) uptake—a key biomarker in DLB diagnosis—could improve diagnostic accuracy. However, DAT imaging is underutilized despite its potential, contributing to delays and suboptimal patient management.

**Methods:**

This review evaluates DLB diagnostic practices and challenges faced within the U.S. by synthesizing information from current literature, consensus guidelines, expert opinions, and recent updates on DaTscan FDA filings. It contrasts DAT SPECT with alternative biomarkers, provides recommendations for when DAT SPECT imaging may be indicated and discusses the potential of emerging biomarkers in enhancing diagnostic approaches.

**Results:**

The radiopharmaceutical ^123^I-ioflupane for SPECT imaging was initially approved in Europe (2000) and later in the US (2011) for Parkinsonism/Essential Tremor. Its application was extended in 2022 to include the diagnosis of DLB. DaTscan’s diagnostic efficacy for DLB, with its sensitivity, specificity, and predictive values, confirms its clinical utility. However, US implementation faces challenges such as insurance barriers, costs, access issues, and regional availability disparities.

**Conclusion:**

^123^I-ioflupane SPECT Imaging is indicated for DLB diagnosis and differential diagnosis of Alzheimer’s Disease, particularly in uncertain cases. Addressing diagnostic obstacles and enhancing physician-patient education could improve and expedite DLB diagnosis. Collaborative efforts among neurologists, geriatric psychiatrists, psychologists, and memory clinic staff are key to increasing diagnostic accuracy and care in DLB management.

## 1 Introduction

Dementia with Lewy Bodies (DLB) is a progressive neurodegenerative disorder characterized by the presence of abnormal brain deposits of alpha-synuclein in the form of Lewy bodies and neurites (1–3). It is the second most common neurodegenerative dementia after Alzheimer’s disease (AD) (4), affecting 1.4 million Americans and accounting for 4–5% of all clinical cases (5), although findings from neuropathological studies provide estimates that range between 15–20% and are accompanied by AD pathology (6–8). Additionally, Lewy pathology is found at autopsy in 8–12% of cognitively unimpaired individuals over age 70 (9–12).

However, despite DLB’s prevalence, awareness among general neurologists and other healthcare professionals is limited (13, 14), and DLB is not always routinely considered in the differential diagnosis of patients presenting with cognitive decline, particularly when accompanied by non-cognitive features such as delirium, confusion, and fluctuations (15). Underdiagnosis is supported by findings from a meta-analysis indicating that 20% of DLB diagnoses based on clinical criteria alone are incorrect, with patients most frequently misdiagnosed with AD (16). This is further bolstered by caregiver reports that 78% of patients with DLB receive other diagnoses before a final clinical diagnosis of DLB (17) and that 1 in 3 cases of DLB are missed (18). Factors that may contribute to this under-recognition include overlapping symptoms with those of other dementias (particularly AD), heterogeneous presentations (e.g., hallucinations, Parkinsonism, autonomic dysfunction, REM sleep behavior disorder, etc.), limited exposure to DLB cases during clinical training, small numbers of DLB specialty centers, or lack of familiarity with how to apply DLB consensus criteria in clinical practice. The most recent diagnostic criteria for DLB underscore the importance of biomarkers, particularly those categorized as indicative or directly related to core clinical features. However, underutilization of these biomarkers may contribute to the under-recognition of DLB.

Indicative biomarkers include reduced dopamine transporter (DAT) uptake in the basal ganglia using Single Photon Emission Computed Tomography (SPECT) or Positron Emission Tomography (PET) imaging, REM sleep behavior disorder (RBD) using Polysomnography (PSG), and reduced uptake on ^123^Iodine-metaiodobenzylguanidine myocardial scintigraphy (^123^I-MIBG) (1), which can assist in differentiating DLB from AD and most other causes of dementias with the exception of Parkinson’s disease (PD) dementia. Notably, the long preclinical period of Lewy body pathology (1) further complicates diagnosis, making these biomarkers particularly valuable for early and accurate diagnosis.

To address the diagnostic challenges in DLB, there is a pressing need to bolster knowledge and application of indicative biomarkers of DLB. Among these, DAT imaging via SPECT and PET holds particular significance due to its ability to assess dopaminergic neurons’ status directly. DAT radiopharmaceuticals used in SPECT include [99mTc]TRODAT-1, [^123^I]PE2I, and ^123^I-ioflupane (also known as [^123^I]FP-β-CIT, or DaTscan^™^, GE HealthCare, United States), and many other tracers [for reviews see; (19, 20)]. ^123^I-ioflupane (DaTscan) is the only FDA-approved agent in the US for distinguishing DLB from AD as of 2022, which marks a significant stride in its clinical applicability. This tracer has also been widely applied in PD since its approval in 2011 for differentiating from essential tremor, underscoring its established efficacy in identifying dopaminergic deficits (21–25).

In PET imaging, tracers like [18F]FP-CIT, [18F]LBT-999, and [18F]FE-PE2I are used, offering additional avenues for assessing dopaminergic neuron integrity (26). Fluorodopa F 18 (27), a fluorinated analog of Levodopa, is another significant diagnostic agent used in PET. As noted by the FDA in 2019, its primary application is in evaluating Parkinsonian syndromes [i.e., PD, Progressive Supranuclear Palsy (PSP), Corticobasal Degeneration (CBD), and Multiple Systems Atrophy (MSA)]. Fluorodopa F 18 PET serves adjunctly with other diagnostic procedures to visualize dopaminergic nerve terminals in the striatum. However, this does not target DAT like DaTscan. Of note, the use of DAT tracers, which has evolved since the 1970s with various ligands and imaging techniques, has greatly advanced our understanding of the pathophysiology and progression of PD. These tracers have been instrumental in confirming dopamine deficiency, a hallmark of PD, aiding in differentiating PD from other movement disorders, and monitoring disease progression (28).

Improvement in diagnosis is crucial for optimizing patient care outcomes, particularly with the ongoing development of new therapeutic options (28, 29). The relatively recent approval of ^123^I-ioflupane for SPECT to assist with diagnosing DLB from AD warrants a closer examination of the role of DAT SPECT imaging in clinical practice and how it can help address current diagnostic challenges for DLB specifically. Thus, the primary objective of this manuscript is to evaluate the practical application of DAT SPECT imaging in an effort to minimize some of the challenges faced by practitioners in diagnosing DLB within the context of the United States (US). We will describe how DAT SPECT is integrated with current diagnostic guidelines and indicative biomarkers for DLB, its interpretative nuances in clinical practice, and its future in advancing diagnostic accuracy for DLB.

### 1.1 The spectrum of Lewy body dementias

Lewy Body Dementia is an umbrella term encompassing DLB and Parkinson’s Disease Dementia (PDD). Both conditions are pathologically characterized by the accumulation of Lewy bodies and neurites (30, 31). While sharing similar pathological underpinnings, DLB and PDD often differ in their initial clinical presentations, progression, and overall clinical course (32, 33).

DLB typically presents with cognitive-psychiatric symptoms from its onset (1). Patients with DLB often exhibit cognitive fluctuations characterized by periods of clarity and episodes of confusion or drowsiness (34). Although memory impairment may manifest early in DLB, deficits in executive function, attention, and visuospatial abilities tend to be more pronounced in the early stages of the disease compared to AD (14, 35). In addition, episodic memory deficits in DLB generally improve with cueing, while episodic memory deficits in AD do not (36, 37). Psychiatric changes in DLB are common and include recurrent and well-formed visual hallucinations, symptoms of depression, anxiety, and apathy, and may be preceded by rapid eye movement sleep behavior disorder (RBD) (38). Motor features, such as bradykinesia and rigidity, may accompany or follow the onset of cognitive-behavioral symptoms but may not be evident in all DLB patients. The consensus criteria for DLB apply a “one-year rule” for the differentiation from PDD (1), which asserts that if cognitive decline leading to dementia occurs before or concurrently within one year of the onset of parkinsonian motor symptoms, the diagnosis is more likely to be DLB. Conversely, if dementia occurs more than one year after the onset of Parkinsonian symptoms, the diagnosis is more likely to be PDD.

PDD typically first manifests with motor features characteristic of PD (e.g., bradykinesia, rigidity, rest tremor). These motor symptoms are usually present for several years before the onset of cognitive symptoms (33). Findings from a recent meta-analysis reported that 15–20% of patients with PD patients developed dementia after five years and 46% at ten years, with this significantly increasing a survival rate greater than ten years (39). The cognitive profile of PDD includes difficulties with attention, processing speed, executive functioning, and visuospatial skills (40). Psychiatric and mood changes, such as apathy, depression, and visual hallucinations, are also observed in individuals with PDD (41).

### 1.2 Clinical practices for DLB diagnosis in the US

The diagnostic criteria for DLB have evolved, culminating with the fourth consensus criteria in 2017 (Table 1) (1). These criteria build upon the core and supportive features established from prior iterations (2, 42, 43). Notably, the 2017 criteria elevated RBD to a core feature, removed the suggestive feature category, and downgraded antipsychotic hypersensitivity to a supportive feature. While this paper focuses on the 2017 criteria, it is acknowledged that some readers may still need to be fully acquainted with these updates, particularly concerning the integration of indicative biomarkers. Therefore, we will provide a brief overview of the role of these biomarkers in establishing a “probable” or “possible” diagnosis of DLB.

**Table 1:** Overview of McKeith (1) criteria for diagnosing Dementia with Lewy Bodies (DLB).

Category	Description
**Essential features**	Progressive cognitive decline of sufficient magnitude to interfere with normal social or occupational functions, or with usual activities. Deficits in attention, executive function and visual spatial ability may be prominent early on.
	Recurrent and well-formed visual hallucinations
**Core clinical features**	REM sleep behavior disorder
	Fluctuations in cognition
	Parkinsonism (one or more cardinal features; i.e., bradykinesia, rest tremor, or rigidity)
	Other hallucinations (e.g., auditory, tactile)
	Systematized delusions
	Depression, anxiety and apathy
**Supportive clinical features**	Symptoms suggestive of orthostatic hypotension Postural instability, repeated falls, syncope, transient episodes of unresponsiveness Constipation
	Urinary incontinence (non-neurological)
	Severe sensitivity to antipsychotic agents
	Symptoms suggestive of orthostatic hypotension
	Hyposmia; hypersomnia

This table provides a summary of the McKeith (1) criteria for the diagnosis of Dementia with Lewy Bodies (DLB), as outlined in the latest consensus report. The table categorizes the diagnostic criteria into three groups: essential features, core clinical features, and supportive clinical features. Essential features include the presence of progressive cognitive decline that significantly impacts social or occupational functioning. Core clinical features are symptoms commonly associated with DLB and contribute to its differential diagnosis, including recurrent visual hallucinations, REM Sleep Behavior Disorder, fluctuations in cognition, and Parkinsonism. Supportive clinical features may not be specific to DLB but can support the diagnosis when present in conjunction with core features. These supportive features encompass a range of physical and psychological symptoms, including but not limited to postural instability, syncope, and severe sensitivity to antipsychotic agents. The overview provided by this table is intended to aid clinicians in recognizing the spectrum of symptoms associated with DLB and should be used in conjunction with comprehensive clinical assessment and judgment.

Indicative biomarkers (Table 2) include reduced DAT uptake demonstrated by SPECT or PET imaging, reduced MIBG on myocardial scintigraphy, and polysomnography (PSG) confirmation of RBD, each reflecting unique pathology underlying core clinical features. Supportive biomarkers include relative preservation of medial temporal lobe structures evident on CT/MRI scans, generalized low uptake on SPECT/PET perfusion/metabolism scans frequently accompanied by reduced occipital activity, preservation of 18Fluorodeoxyglucose PET uptake in the posterior cingulate “cingulate island sign” (52), and the presence of prominent posterior slow-wave EEG activity displaying periodic fluctuations in the pre-alpha/theta range. While these supportive biomarkers can assist in diagnosis, they lack specificity for DLB.

**Table 2:** Summary of indicative and supportive biomarkers for DLB.

	Relevance	U.S. regulatory status	U.S. availability	Sensitivity	Specificity	Studies
**Indicative biomarkers**						
DaTscan ([^123^I] FP-CIT SPECT):	As a visualizing agent for the dopamine transporter in the brain, DaTscan provides a concrete method to distinguish DLB from other dementia types, especially Alzheimer’s Disease	FDA approved for use as as an adjunct to other diagnostic evaluations On-label for DLB	Most often performed in Freestanding Imaging Center	78–93% (using visual assessment)	84–90%	Thomas et al. (44), Kamagata et al. (45) and Tiraboschi et al. (46)
Cardiac [^123^I] MIBG scintigraphy	reflects post-ganglionic sympathetic cardiac innervation, which is reduced in DLB. Calculated by heart to mediastinum (H/M) ratio	Off-label for DLB, not FDA approved	Limited and primarily used in research settings	70.0% for early H/M ratio., 80.0% for delayed H/M ratio, 80.0% for washout rate	96.2% specificity for early H/M ratio., 92.3% for delayed H/M ratio, 84.6% for washout rate	Matsubara et al. (47)
Polysomnography (PSG) confirmation of REM sleep without atonia	Assesses REM sleep behavior disorder, a common symptom in DLB patients	Off-label for DLB diagnostic purposes but is a standard tool for sleep disorders	Widely available	85–93%	83–99%	Puligheddu et al. (48)
**Supportive biomarkers**						
Relative preservation of medial temporal lobe structures on CT/MRI scan	Relative preservation of medial temporal lobes suggests a lower likelihood of AD and supports a DLB diagnosis	FDA-approved for standard clinical dementia evaluations but is used off-label for specific DLB differentiation	Widely available	64%	68%	Harper et al. (49)
Generalized low uptake on SPECT/PET perfusion/metabolism scans with reduced occipital and/or the “posterior cingulate island sign” on FDG-PET imaging	Signs of occipital hypometabolism and posterior cingulate island (high ratio of glucose metabolism in posterior cingulate versus precuneus and cuneus) are supportive of a DLB diagnosis	FDA-approved for differentiating types of dementia but is used off-label specifically for DLB diagnosis	Accessible in U.S. facilities with advanced imaging capabilities	70%	74%	O’Brien et al. (24) and Lim et al. (50)
Prominent posterior slow-wave EEG activity with periodic fluctuations in the pre-alpha/theta range	Distinct EEG patterns suggest DLB, especially with noted fluctuations in the pre-alpha/theta range. Delta activities in pseudo periodic patterns, that in combination have a predictive power of >90% for DLB compared to AD	FDA-approved for neurological evaluations	Widely accessible	72–79% (overall GTE)	76–85% (overall GTE)	Law et al. (51)

This table details the indicative and supportive biomarkers for Dementia with Lewy Bodies (DLB), outlining their relevance, regulatory status in the U.S., and availability. “DaTscan ([^123^I] FP-CIT SPECT)” = Dopamine Transporter Scan using Iodine-123 fluoropropyl-Carbomethoxyiodophenyltropane Single Photon Emission Computed Tomography. “Cardiac [^123^I] MIBG scintigraphy” = Iodine-123 metaiodobenzylguanidine scintigraphy. “PSG” = Polysomnography. “CT/MRI scan” = Computed Tomography/Magnetic Resonance Imaging. “SPECT/PET perfusion/metabolism scans” = Single Photon Emission Computed Tomography/Positron Emission Tomography scans. “EEG” = Electroencephalography, AD = Alzheimer’s Disease, RBD = REM behavior sleep disorder, GTE = Grand Total EEG score.

A “probable” DLB diagnosis is determined by either two or more core clinical features of DLB or one core clinical feature coupled with one or more indicative biomarkers. Core clinical features typically include fluctuating cognition, visual hallucinations, RBD, and Parkinsonian motor symptoms. These indicative biomarkers significantly enhance the specificity of the diagnosis, particularly when clinical features alone might be insufficient for a definitive diagnosis. A “possible” DLB diagnosis, on the other hand, is considered when only one of these core clinical features is present without any indicative biomarker evidence or when indicative biomarkers are present but without the core clinical features. However, diagnosing DLB should not be based on biomarkers alone (1), and a definitive diagnosis of DLB relies on autopsy neuropathological confirmation.

While myocardial scintigraphy using MIBG is a valuable indicative biomarker for DLB, its usage in the US is less common, primarily due to availability and expertise constraints (53). Moreover, this has not been approved by FDA for use in this indication. Alternatively, PSGs are typically only performed following sleep complaints. Thus, of the indicative biomarkers available in the US, DAT SPECT imaging is most likely to have a key role in diagnosing DLB.

### 1.3 Importance of accurate and early diagnosis of DLB

An accurate diagnosis ensures patients receive the most appropriate and effective treatments, minimizing potential side effects and maximizing efficacy (13). This aspect is essential considering the heightened sensitivity of patients with DLB to various antipsychotic medications and anti-dopaminergic agents (i.e., prochlorperazine), which can exacerbate symptoms or lead to severe adverse reactions (54, 55).

While disease-modifying therapies (DMTs) for DLB are not yet available, early diagnosis is essential to start interventions that may slow symptom progression, alleviate discomfort, and improve quality of life (5). For example, early initiation of cholinesterase (ChE) inhibitors, which inhibit the enzyme acetylcholinesterase to increase the availability of acetylcholine and enhance cholinergic transmission, has shown considerable efficacy in managing DLB, especially given the cholinergic deficits in this condition. These benefits extend beyond cognitive improvement to potentially ameliorating behavioral (sleep disturbances) and psychiatric symptoms (hallucinations). Potentially slowing symptom progression leading to enhanced overall patient and caregiver reported outcomes and quality of life (13, 56).

Moreover, an early and precise diagnosis provides essential clarity for patients and their families, enabling informed decision-making regarding care, facilitating access to support services, and managing expectations. It also ensures that patients are referred to occupational therapy (OT) and physical therapy (PT) early, which may be more crucial in DLB than in AD (57, 58). From a research perspective, accurate diagnosis is necessary for enrolling the target participant in clinical trials, to ensure the accurate evaluation of therapeutic agents (14).

Additionally, receiving a correct diagnosis avoids the frustration and feelings of being misguided that can arise when diagnoses are later revised from AD to DLB or other conditions. This not only impacts patient trust but also significantly influences their treatment path and family’s understanding of the disease.

### 1.4 Challenges of diagnosing DLB clinically

Diagnosing DLB poses challenges, particularly in non-specialized settings. Recognizing DLB is more straightforward in specialized clinics—like those focusing on memory, cognitive disorders, or movement disorders—where there is greater expertise in differentiating DLB from other common neurodegenerative conditions, such as AD or PD, which often present with overlapping symptoms. In general practice, however, variability in DLB clinical presentation coupled with limitations in diagnostic tools can hinder accurate diagnosis.

Beyond AD and PD, DLB must also be differentiated from other conditions, such as frontotemporal degeneration with Parkinsonism, vascular cognitive impairment, and posterior cortical atrophy. The latter, characterized by prominent visuospatial and visuoperceptual abnormalities, can result from DLB but is also seen in AD, corticobasal syndrome, and Creutzfeldt-Jakob disease (59, 60). This wide array of potential clinical diagnoses can lead to misdiagnosis or delayed diagnosis.

In a study of DLB care partners (61), the diagnosis of DLB can be delayed as much as 18 months, with most patients seeing more than three physicians over multiple visits. Misdiagnosis was common, with 78% receiving a diagnosis other than DLB, often depending on the presenting symptoms and the first provider encountered (e.g., movement disorder or cognitive behavioral neurologist, general neurologist, sleep specialist, psychiatrist, geriatrician, gastroenterologist). Common misdiagnoses included other forms of dementia, such as AD (26%), or related movement disorders, such as PD (39%). In another study, findings revealed that when behavioral symptoms such as visual hallucinations, delusions, and mood disorders are predominant, then a psychiatric diagnosis was made in 24% of cases (17). Alarmingly, about 22% of patients did not receive a diagnosis when first presenting to their physician. This lack of diagnosis occurred most frequently when patients first presented these symptoms to primary care physicians, who may lack the specialized training to recognize the early signs of DLB (61). In the instances where a diagnosis of DLB was eventually made, 62% of these diagnoses were made by neurologists. Other specialists involved in diagnosing DLB included psychiatrists (9%), geriatricians (8%), psychologists (8%), and, to a lesser extent, primary care physicians, comprising family physicians (5%) and internal medicine.

## 2 Understanding DAT SPECT results in DLB (^123^I-ioflupane SPECT use in DLB)

Diminished uptake of the DAT tracer in the basal ganglia, identified through ^123^I-ioflupane SPECT imaging, is an indicative biomarker of DLB (1). Tracer uptake reflects the loss of nigro-striatal network, a phenomenon similarly observed in PD. The efficacy of ^123^I-ioflupane SPECT in detecting DLB has demonstrated a sensitivity of 80% and specificity of 92% (18). This research supported DAT SPECT imaging as Class I evidence for DLB diagnosis. However, while generally high, its specificity necessitates careful interpretation in the context of overlapping clinical features with other Parkinsonian syndromes.

The positive predictive value of ^123^I-ioflupane SPECT is 82.4%, while the negative predictive value is 87.5% (62). Therefore, while a positive scan is highly suggestive of DLB, it does not exclude other Parkinsonian syndromes characterized by nigrostriatal degeneration. Conversely, a negative DAT SPECT result substantially lowers the probability of DLB. Yet, it is important to consider that up to 10–20% of DLB patients may present with a normal DAT SPECT result at the baseline assessment (44). One possible explanation for this discrepancy may be that alpha-synuclein pathology may not have affected the substantia nigra and might be limited to cortical or limbic regions. Nevertheless, combining DAT SPECT results with clinical assessments is important to form a comprehensive diagnostic approach, as a negative result does not categorically exclude DLB.

### 2.1 Qualitative (visual) DAT SPECT interpretation

Qualitative, or visual, assessment remains the standard practice for interpreting DAT SPECT imaging results. This approach is consistent with the current EANM practice guideline/SNMMI procedure standard for dopaminergic imaging in Parkinsonian syndromes (20). Though interpreting a DaTscan image is relatively straightforward, formal image reading and its reporting are typically generated by nuclear medicine radiologists.

An abnormal DAT tracer uptake is typically characterized by either:

1. Putamen predominant loss of activity at the striata often with asymmetry (“comma-shaped” striatum loses signal predominantly at the putamen posteriorly and becomes more “dot-shaped”),
2. More uniform or balanced loss of activity across the striata (“comma-shaped” striata are broadly maintained, but the degree of activity is reduced, resulting in a loss of contrast with the background, sometimes called “weak commas”).

Note: Almost absent activity at the striata is sometimes known as a “burst striatum” appearance.

**Figure 1:**
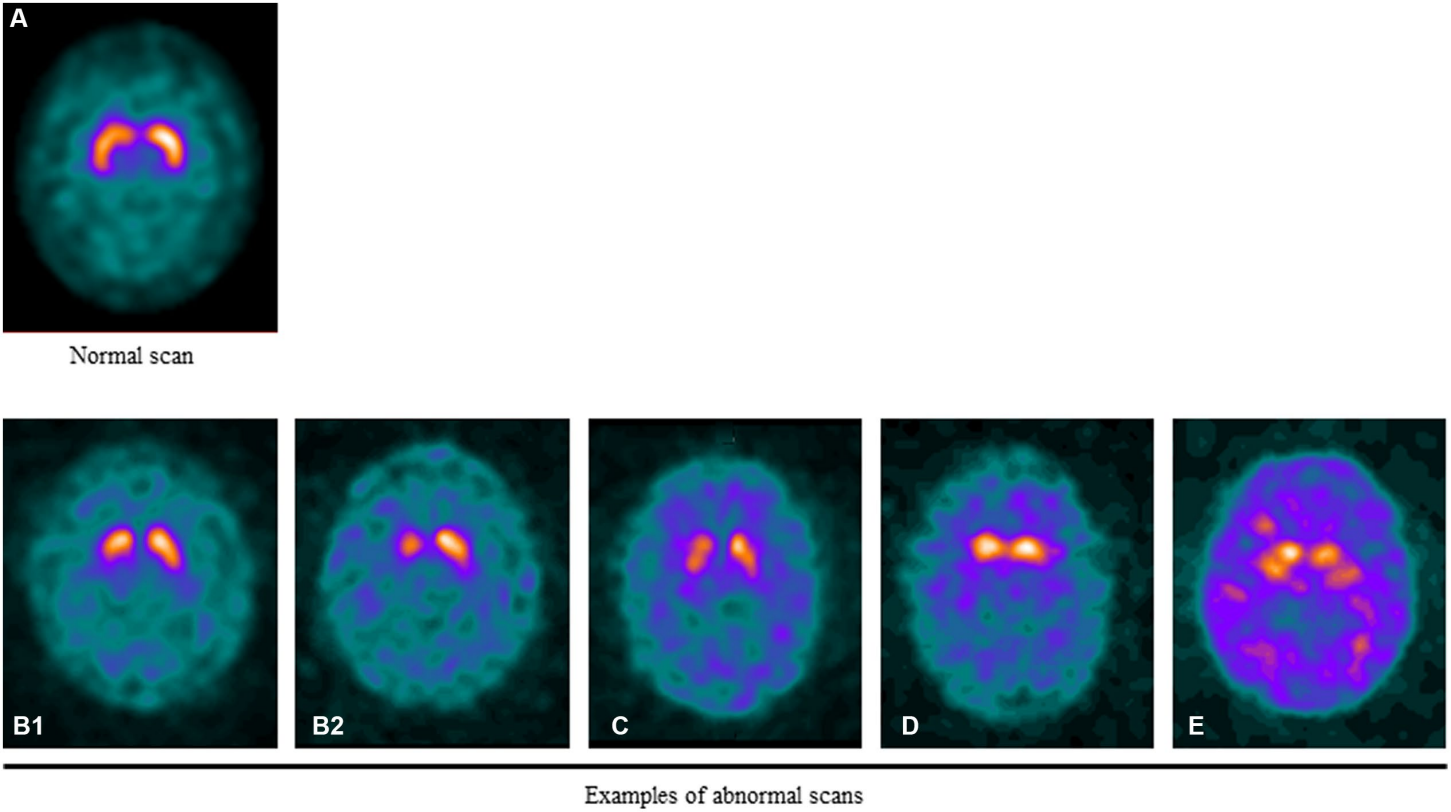
Examples of normal and abnormal DAT SPECT scans. **(A)** DAT signal in a healthy individual. Note the symmetric uptake of the tracer in the caudate and putamen that appears “comma-shaped”. **(B1)** Mild asymmetric putaminal reduction. **(B2)** Moderate/severe asymmetric putaminal reduction. Marked loss of activity in the putamen. **(C)** Balanced loss across the striata with relatively raised background activity. **(D)** Bilateral loss of DAT signal in the putamen with relative preservation in the caudate resulting in a “dot-shape” or “egg-shape” appearance. **(E)** Very little DAT signal at the striata evident by the marked background activity relative to the striatum sometimes known as “burst striata”.

Examples of normal and abnormal scans are shown in Figure 1.

While the primary clinical assessment involves a qualitative evaluation that categorizes the scan as positive or negative, specific protocols, as outlined in the Society of Nuclear Medicine (SNM) (63), incorporate a more nuanced, semi-quantitative analysis. This includes assessing the degree of uptake reduction and its distribution pattern, which can provide further insight into the severity and progression of dopaminergic deficits. Recently, most of the NM departments in the US have used semi-quantitative software named DaTQUANT^™^ (GE Healthcare), which enables the sharing of DAT image intensity measurement figures. This is done as an adjunct to the visual read.

The qualitative interpretation of DAT SPECT imaging is valuable for its potential to substantiate the clinical presentations seen in patients, providing an imaging-based confirmation of symptomatic observations. However, this method is inherently subjective, relying on the expertise of trained observers. Consequently, subtle changes in DAT density, especially in cases with borderline visual interpretations, may be overlooked or with a more balanced reduction at the striata that can be more challenging to perceive. This limitation underscores the value of incorporating semi-quantitative methods in analyzing DAT SPECT images. Findings from a study by Booij et al. (64) demonstrated that combining semi-quantification into the interpretation of ^123^I-ioflupane SPECT scans enabled readers with limited experience to achieve a diagnostic accuracy comparable to that of experienced readers.

It is further noted that imaging patients with dementia and parkinsonian motor features presents specific challenges, such as motion artifacts that can adversely affect image quality and interpretation accuracy (65). These artifacts can significantly impact image quality and interpretation accuracy. To mitigate this, NM guidelines recommend specialized scanning protocols to reduce movement and other potential artifacts, enhancing the reliability of the scan results (20, 63).

## 3 Semi-quantitative assessment

While visual assessment is currently standard, there is growing interest in performing semi-quantitative analysis in DAT SPECT interpretation as an adjunct to visual assessment. Semi-quantification of the DAT SPECT signal measures the signal-to-background ratio at the striatum, including the posterior putamen, an area of the striatum most sensitive to neurodegeneration (66). It also provides a numerical assessment for more balanced loss, which can be challenging to interpret when a minimal change in the shape of the striata is present. This is particularly important in diseases like DLB, where early detection can significantly impact patient management (20). Artifacts commonly encountered in nuclear medicine and molecular imaging, as described in detail in the joint EANM/SNMMI/ANZSNM guidelines (67), offer valuable insights for a broad spectrum of imaging modalities, including DAT SPECT. While not specific to DAT SPECT, these guidelines provide crucial information that can assist neurologists and nuclear medicine specialists in interpreting complex cases. Understanding such artifacts is essential for identifying semi-quantitative measures that may detect more subtle changes indicative of neurodegenerative processes at an earlier stage than visual assessment alone.

Several software tools with FDA clearance are used for semi-quantitative analysis, including MIMneuro^®^ (MIM Software Inc.) and Syngo^®^.via (Siemens Healthineers) and DaTQUANT^™^ (GE Healthcare) being the most widely used. Other non-FDA-cleared software tools include Hermes BRASS^™^ (Hermes Medical Solutions) and BasGanV2. Finally, xSPECT Quant^™^ (Siemens Healthineers) is FDA-cleared for fully quantitative image analysis MIMneuro^®^, Syngo^®^.via, and Hermes BRASS^™^ also offer options for fully quantitative image analysis (68).

Although in the current context, DAT SPECT imaging is unable to reliably differentiate between parkinsonism disorders, however emerging research suggests the future potential. For instance, Iwabuchi et al. (69) demonstrated the effectiveness of semi-quantitative indices from DAT SPECT and MIBG scintigraphy in differentiating various parkinsonian syndromes, particularly highlighting the capacity to distinguish PD from DLB. Their study illustrates the potential of using specific quantitative metrics, like the striatal binding ratio and putamen-to-caudate ratio, to enhance diagnostic accuracy in the context of parkinsonian disorders.

In another more recent study, Ishizawa et al. (70) voxel-based analysis was used to examine striatal DAT binding patterns. Findings revealed that DAT binding was reduced in the posterior striatum in PD and PDD patients, in contrast to similar DAT binding levels observed in MSA-P, PSP, and DLB. Additionally, in PD and PDD, an inverse correlation was found between DAT binding in the anterior striatum and the severity of parkinsonism, a correlation that was not observed in DLB. This study indicates that striatal DAT binding patterns might reflect distinct pathological processes in DLB compared to PDD, suggesting a potential avenue for differential diagnosis using DAT SPECT imaging.

While the promise of semi-quantification is substantial, their clinical application is still in the nascent stages. With advances in imaging technologies, including high-performance CZT SPECT cameras and the development of high-resolution DAT PET tracers, the use of semi-quantification may become a part of future clinical practice.

### 3.1 Clinical and radiologic integration

Integrating clinical and radiologic expertise is fundamental in neurology, particularly for interpreting DAT SPECT imaging in DLB diagnosis. Neurologists and related practitioners must skillfully combine DAT SPECT scan results with the patient’s clinical symptoms, requiring a detailed review of the scans and effective communication of findings to patients and caregivers. Highlighting this, recent studies by Bega et al. (25) and Isaacson et al. (71) have shown the significant impact of DAT SPECT on clinical decision-making. These studies reveal that DAT SPECT imaging not only influences treatment strategies in 54% of patients but also a modification in diagnosis in about 31% of patients, particularly in differentiating between nigrostriatal dopaminergic degeneration and non-nigrostriatal degeneration in patients exhibiting ambiguous parkinsonian signs, thereby improving diagnostic precision in complex clinical situations.

In clinical and radiologic integration, the interpretation of the DAT SPECT imaging is closely coordinated with the patient’s clinical symptoms. For instance, in early PD, an asymmetric reduction in tracer uptake, particularly in the left putamen compared to the caudate nucleus (especially the posterior putamen), is often observed (66). In contrast, a more symmetric putaminal loss assessed with DAT SPECT is frequently observed in DLB and other atypical parkinsonian syndromes such as Multiple System Atrophy (MSA) and Progressive Supranuclear Palsy (PSP) (64). Additionally, these syndromes typically manifest with early involvement of the caudate nuclei, leading to attenuation of the striatum’s “comma” shape. This is due to simultaneous changes in the caudate nucleus and the putamen, which compromise the distinctive anatomy of these regions on imaging.

In visual readings of DAT SPECT images, distinguishing between putaminal and caudate involvement is standard practice. It is generally observed that putaminal loss is suggestive of dopaminergic deficits, while early caudate involvement may indicate atypical Parkinsonian syndromes. However, these observations should be approached with caution in a clinical setting. Individual scan interpretations can vary, and the presence or absence of a caudate signal does not have a universally established diagnostic value. Therefore, DAT SPECT imaging is primarily used to determine the presence or absence of dopaminergic deficits rather than ascertaining differential diagnosis based on specific regional involvement.

### 3.2 Timing of DAT imaging: implications for diagnosis and prognosis

Evidence suggests that DAT imaging can aid in early diagnosis and prognosis. For example, Pasquini et al. (72) study on PD found that early caudate dopaminergic denervation was predictive of an increased risk of cognitive impairment, depression, and gait problems, underscoring the potential predictive value of DAT scans in identifying patients at risk for rapid disease progression. These findings are broadly consistent with findings by Yamamoto et al. (73), which showed a correlation between longitudinal changes in DAT uptake and progression of parkinsonism and cognitive symptoms in DLB. However, their study noted an inverse correlation between the progression of parkinsonism severity and DAT uptake changes in the left posterior putamen, differing from the patterns observed in PD. This distinction suggests that DAT scans may not uniformly predict symptom progression across disorders, reflecting diverse underlying pathophysiological mechanisms between PD and DLB.

An important consideration, however, is the timing of DAT scans in later clinical stages as well as different clinical presentations (74–78). In the Van der Zande et al. (74) study, the authors reported that for some cases where the initial scans showed no apparent dopaminergic deficits, these later evolved into clearly visible dopaminergic deficits, suggesting that early negative results may not preclude the development of dopaminergic deficits as the disease progresses.

In a 4-year longitudinal study of PD patients (77), motor symptom progression was associated with reduced DAT binding across all regions in PD, particularly in the putamen. Furthermore, the findings showed a more significant reduction in ipsilateral putamen than in contralateral putamen, suggesting a pattern that could help assess disease asymmetry and progression. However, a “floor effect” by year 4 indicates the diminishing utility of DAT scans to evaluate disease progression in later stages. In another study on PD (78), the progression of non-motor symptoms (NMS) was only weakly correlated with DAT binding across, suggesting that NMS may predominantly arise from non-dopaminergic pathways. This highlights the limitations of DAT imaging in cases where NMS are most pronounced.

In sum, the variability in the timing of DAT scan changes relative to symptom onset in DLB complicates the diagnostic process, potentially leading to initial misdiagnosis or delayed diagnosis. Moreover, the lack of direct correlation between DAT imaging findings and NMS progression underscores the need for a multimodal diagnostic approach beyond dopaminergic neuron integrity assessment. Clinicians should consider the timing of DAT scans relative to disease onset, the presence of both motor and non-motor symptoms, and the potential for disease progression despite initial negative imaging results. This approach emphasizes the importance of longitudinal monitoring and a multimodal diagnostic strategy incorporating DAT imaging and comprehensive clinical assessments.

### 3.3 Medications that can affect ^123^I-ioflupane SPECT uptake

Before ordering a DAT imaging, it is essential for clinicians to review the patient’s concurrent medication usage carefully. Certain medications can significantly alter DAT tracer uptake, thus impacting the visual interpretation of the scan. Key classes of substances that can interfere include psychostimulants (such as cocaine and amphetamines), certain antidepressants, and specific antipsychotic medications. These can lead to false abnormal or, rarely, false normal interpretations, which are critical considerations for practitioners relying on visual assessments of ^123^I-ioflupane SPECT scans (79). Misinterpretations run the risk of misdiagnoses and, consequently, inappropriate treatment approaches. Note that the use of cholinesterase inhibitors, a first line of treatment for DLB does not appear to affect ^123^I-FP-CIT uptake on SPECT imaging (80).

The recent systematic review (79) builds upon foundational work in this area, such as the review by Booij and Kemp (81), further elucidating the effects of these medications and substances on the visual assessment of DAT SPECT imaging, This ongoing research is critical for guiding practitioners in everyday clinical practice. Additionally, contraindications for DAT SPECT, including hypersensitivity to any components and considerations during pregnancy, must be carefully evaluated. Clinicians are advised to consult the prescribing information for detailed guidelines.

While we focus on medications frequently prescribed to elderly patients due to their prevalence in this demographic, the interaction of these medications with DAT SPECT is also pertinent in younger patients being evaluated for DLB. Understanding these interactions is essential for accurate interpretation and practical clinical application of DAT imaging. Below, we list medications commonly prescribed in the context of DLB evaluation, highlighting those often used in older patients. For a more extensive review of medication interactions with DAT SPECT, we refer to the systematic review by Chahid et al. (79). Table 3 integrates key findings from the Chahid et al. review with additional details on the mechanism of action and clinical uses of various medications relevant to DLB, offering a comprehensive overview of pharmacological considerations and their implications for DAT SPECT imaging.

**Table 3:** Pharmacological considerations in DLB: drug classes, uses, mechanisms, and DAT SPECT imaging implications.

Drug Class	Treatment use	Mechanism of action	Effect on DAT imaging	Days to stop before DAT scan*
***Often used in patients evaluated for DLB***				
Levodopa/Carbidopa	Motor symptoms in DLB	Increases dopamine levels	Little to no effect	–
Dopamine agonists	Stimulate dopamine receptors	Mimics dopamine effect	Little to no effect	–
MAO-B inhibitors	Increase dopamine availability	Inhibits monoamine oxidase-B enzyme	Little to no effect	–
COMT inhibitors	Prolong effect of levodopa	Inhibits catechol-O-methyltransferase enzyme	Little to no effect	–
Amantadine	Mild dopaminergic effects reduce dyskinesia	Modulates glutamate receptors, increases dopamine release	Little to no effect	–
SSRIs	Depression and anxiety in DLB	Binds to the serotonin transporter	Slight (~10%) signal-to-noise ratio enhancement.	–
SNRIs	Depression and anxiety in DLB	Inhibit the reuptake of serotonin and norepinephrine	Insufficient data /little to no effect	–
Amitriptyline (Tricyclic Antidepressant)	Depression, often avoided in DLB	Inhibiting the reuptake of serotonin and norepinephrine/anticholinergic	Insufficient data/ little to no effect	–
Bupropion (Antidepressants)	Depression in PD and DLB	Norepinephrine-dopamine reuptake inhibitor	Can result in a false positive scan	5–8 days
Haloperidol (Antipsychotics)	Psychosis management	Competitively blocks post-synaptic dopamine (D2) receptors	Can result in a false positive scan	5 days
Fentanyl (Opioids)	Pain management	Binds to opioid receptors	Can result in a false positive scan	2–5 days
Codeine (Opioids)	Pain management	Binds to opioid receptors	Can result in a false positive scan	1–2 days
Dexamphetamine (Stimulants)	ADHD, wakefulness	Norepinephrine and dopamine reuptake inhibitor	Can result in a false positive scan	1–7 days
Methylphenidate	ADHD, wakefulness	Norepinephrine and dopamine reuptake inhibitor	Can result in a false positive scan	1–7 days
Dexmethylphenidate	ADHD, wakefulness	Norepinephrine and dopamine reuptake inhibitor	Can result in a false positive scan	1–7 days
Modafinil/Armodafinil	Excessive daytime sleepiness in DLB	Increases dopamine and norepinephrine	Can result in a false positive scan	3 days
***Substances of abuse***				
Methamphetamine		Increase dopamine availability	Can result in a false positive scan	1–7 days
Cocaine		Binds to DAT	Can result in a false positive scan	1–2 days
*Other drugs known to interfere with ^123^I-ioflupane*				
Phentermine	Weight loss	Increases norepinephrine, dopamine, and serotonin	Can result in a false positive scan	1–5 days
Ephedrine	Hypotension perioperatively	Alpha and beta-adrenergic agonist	Can result in a false positive scan	1 day

This table summarizes various drug classes and their implications in Dementia with Lewy Bodies (DLB) management. *Assumes that drug interactions with DAT imaging are reversible and concentration-dependent. Stopping the medication at least five times the half-life before DAT imaging is generally recommended. However, the normalization of DAT binding post-discontinuation may take longer in some instances, such as those involving drug interactions that inhibit drug metabolism or in individuals with specific genotypes (e.g., poor metabolizers). This should be taken into account when scheduling and interpreting DAT imaging results [adapted from Chahid et al. (79)]. Levodopa is often combined with Carbidopa (Carbidopa/Levodopa) to enhance efficacy. Monoamine Oxidase-B (MAO-B) Inhibitors and Catechol-O-Methyltransferase (COMT) Inhibitors are mentioned for their specific mechanisms of action. Selective Serotonin Reuptake Inhibitors (SSRIs) and Serotonin-Norepinephrine Reuptake Inhibitors (SNRIs) are discussed for their impact on depressive symptoms in DLB. Attention-Deficit/Hyperactivity Disorder (ADHD) is noted in the context of stimulant use. Days to stop before Dopamine Transporter (DAT) SPECT imaging are provided where relevant.

### 3.4 General considerations for medication management in DLB

#### 3.4.1 Anticholinergic medications

Anticholinergic medications are often used in PD to reduce motor symptoms like tremors and are generally not recommended for patients with DLB. These drugs work by inhibiting the action of acetylcholine, a neurotransmitter that plays a key role in cognitive functions, which are often already impaired in DLB (33, 82, 83). Patients with DLB are sensitive to the side effects of anticholinergic medications, which include confusion, memory loss, hallucinations, and worsening of cognitive deficits. Some examples of these medications include low-potency neuroleptics (e.g., thioridazine), tricyclic antidepressants (e.g., amitriptyline), antiparkinsonian anticholinergics (e.g., trihexyphenidyl), and antispasmodics for bladder or gastrointestinal issues. Physical side effects like urinary retention, constipation, and dry mouth can further complicate the clinical management of DLB. Given these considerations, clinicians must exercise caution in prescribing anticholinergic medications to DLB patients, not only due to their adverse cognitive and neurological effects but also because of their potential impact on the interpretation of DAT scans. For example, the exacerbation of cognitive impairment by anticholinergics could lead to misinterpretation of the progression of DLB when correlating clinical symptoms with DAT findings. Alternative treatments with a more favorable side effect profile should be sought for managing symptoms in DLB.

In managing the complex pharmacotherapy of DLB, collaboration with healthcare providers is essential. Clinicians, patients, and caregivers are advised to consult with their healthcare team regularly. This ensures a comprehensive treatment approach, considering the latest research findings, medication interactions, and individual patient responses. Ultimately, the benefits and risks of stopping a medication that may interfere with the uptake of DaTscan is a clinician’s decision that should be made individually after evaluating the patient’s risks and benefits.

### 3.5 Selective serotonin reuptake inhibitors

These medications are often prescribed for depression and anxiety symptoms in DLB. They might slightly enhance the signal-to-noise ratio of DAT SPECT, but according to the guidelines (20), it is not recommended to withdraw SSRIs before conducting DAT imaging in routine clinical practice. This recommendation is based on the understanding that while SSRIs may influence striatal ^123^I-ioflupane binding ratios quantitatively, the extent of this effect is relatively small, approximately 10%, mainly due to ^123^I-ioflupane binding to the serotonin transporter outside of the striatum. The review by Chahid et al. (79) provides further insights into SSRI interference studies, reinforcing the notion that these effects, although present, are minimal and should not significantly impact the interpretation of visual assessments in clinical routine application. However, in the context of research studies where precision and quantitative measures are more critical, the potential influence of SSRIs on DAT imaging results should be considered.

## 4 Diagnosing DLB versus AD using DAT SPECT

The most common diagnostic challenge that general neurologists encounter is differentiating between the early clinical presentations of DLB and AD, given their clinical cognitive and behavioral symptom overlap (84). This is especially true in cases where other hallmark features of DLB – such as hallucinations, Parkinsonism, autonomic dysfunction, and RBD – are as apparent. For example, where the clinical presentation might predominantly feature psychiatric symptoms, including delirium, forgetfulness, confusion, or personality changes, thereby resembling AD and suggesting the possibility of mixed pathology.

AD is characterized by the presence of amyloid-beta (Aβ) plaques and tau pathology, with estimates suggesting that comorbid AD pathology occurs in about 48–88% of DLB cases (8, 85–89) while up to 60% of AD patients will have comorbid Lewy body pathology (90).

The presence of concomitant AD pathology in DLB may contribute to the variability of clinical presentations, although their distributions may differ between conditions. For example, patients with DLB who also harbor a significant burden of neocortical Alzheimer’s pathology might exhibit an amnestic syndrome, which is more typical of AD than DLB. Alternatively, a patient with posterior cortical atrophy can present with predominantly visuoperceptual abnormalities, which is more typical of DLB than AD.

In these complex scenarios, DAT SPECT emerges as a valuable tool by identifying dopaminergic neuron loss, indicative of DLB but not typically present in AD (91). Additionally, the DLB diagnostic criteria also recommend the use of Magnetic Resonance Imaging (MRI). MRI can provide further distinction by revealing structural brain changes that differentiate DLB from AD and healthy controls. For instance, MRI may show specific patterns of atrophy or other neuroimaging markers that are more characteristic of one condition over the other. Therefore, a combination of DAT SPECT and MRI can offer a more comprehensive assessment, aiding clinicians in accurately diagnosing DLB. This multimodal approach aligns with current DLB criteria and enhances diagnostic confidence, especially in cases where clinical symptoms alone are insufficient for a definitive diagnosis.

## 5 Other scenarios warranting a DAT SPECT

Although a core feature of DLB, fluctuations can occur in AD (~20%) and healthy controls (92, 93). Cognitive fluctuations are characterized by spontaneous alterations in alertness and attention that can manifest as excessive daytime somnolence, confused or illogical train of thought, staring spells, or blank looks that can last seconds to hours (94). Cognitive fluctuations can be challenging to diagnose and may be confused for partial complex seizures or other epileptiform activity, medication effects, or metabolic derangements. A patient presenting with dementia and also cognitive fluctuations can be diagnosed with DLB based on their clinical symptom (cognitive fluctuations = one core feature) and DAT SPECT (abnormal DAT SPECT = one indicative biomarker). Similarly, for a patient with dementia presenting with a psychiatric feature as a core feature (e.g., visual hallucination, misidentification delusions), an abnormal DAT scan can confirm a diagnosis of DLB. Moreover, by identifying DLB through DAT imaging, clinicians can make informed decisions to select antipsychotic medications cautiously, preferably opting for those with minimal dopamine-blocking effects, such as Quetiapine, as mentioned previously.

DAT imaging can reliably reveal reduced DAT levels in DLB patients, however, it is not an unequivocal marker. A subset of clinically diagnosed probable DLB individuals may have DAT SPECT results (18, 95, 96). Given these considerations, DAT imaging should be part of a comprehensive diagnostic approach in DLB, combined with clinical assessment.

### 5.1 Case example 1: probable DLB with subtle bradykinesia but no other parkinsonism features

Here, an abnormal DAT SPECT can support a DLB diagnosis in alignment with DLB consortium guidelines.

- A 72-year-old man presents with a 2-year history of cognitive decline and behavioral issues. Cognitive testing reveals difficulty with word recall, which improves with recognition/cueing, executive function tasks, visual construction (i.e., clock drawing), and fluctuating attention. Mild mood changes (e.g., depression, apathy) are also present. On physical exam, there is generalized psychomotor slowing but no specific Parkinsonian features. Given the non-amnestic cognitive presentation, fluctuations, and behavioral changes, a diagnosis of probable DLB is suspected clinically, and a DAT SPECT is ordered to assist with the diagnosis.

#### Scenario 1: DAT SPECT shows dopaminergic deficit

##### Findings

Reduced DAT uptake in the basal ganglia, more pronounced in the putamen.

##### Interpretation

This result supports the diagnosis of DLB. The reduced DAT uptake, particularly in the absence of classic Parkinsonian signs, aligns with the neuropathological changes typically seen in DLB.

##### Impact on management

This would validate the clinical suspicion of DLB and guide the treatment towards DLB-specific management. In light of the cognitive symptoms and fluctuating attention observed, initiating cholinesterase inhibitor therapy could offer significant benefits, as these agents are known to improve cognitive function and may help with behavioral issues in DLB patients. Importantly, given the heightened sensitivity of DLB patients to antipsychotic medications, which can exacerbate symptoms or lead to severe adverse reactions, there should be a heightened caution in their use.

#### Scenario 2: DAT SPECT is normal (SWEDD)

##### Findings

Normal DAT uptake in the striatal regions.

##### Interpretation

A normal DAT SPECT suggests that dopaminergic neurodegeneration may not be the underlying cause of the patient’s symptoms. This indicates that the patient’s cognitive and behavioral symptoms have a different etiology than DLB.

##### Impact on management

This would prompt a re-evaluation of the diagnosis, potentially exploring other causes of cognitive impairment such as Alzheimer’s disease, frontotemporal dementia, or non-neurodegenerative conditions. It could also lead to a reconsideration of treatment strategies.

### 5.2 Case example 2: differentiation from DLB from AD

- An 81-year-old woman presents with a 3-year history of progressive memory decline. Following a brief hospitalization, she experienced an episode of delirium and hallucinations, which resolved over three days. No parkinsonism was found on the exam. Mental status testing demonstrated a moderate episodic memory impairment without benefits from cueing, mild deficits in orientation to date and day of the week, mild executive dysfunction, and impaired clock drawing. The family is interested in treatment with monoclonal antibodies, but it is difficult to differentiate between DLB and AD clinically. A DAT SPECT is ordered to assist with the diagnosis and treatment decisions.

#### Scenario 1: DAT SPECT indicates dopaminergic deficit

##### Findings

Reduced DAT uptake in the basal ganglia, especially in the putamen.

##### Interpretation

This result suggests the presence of a dopaminergic deficit, more consistent with DLB than AD. The hallmark of DLB involves a reduction in dopamine transporter availability in the striatum, which this DAT SPECT result would support.

##### Impact on management

Confirmation of DLB would lead to reconsidering treatment options, possibly moving away from AD-specific treatments like monoclonal antibodies and towards DLB-targeted therapies such as cholinesterase inhibitors to improve cognitive and psychiatric symptoms.

#### Scenario 2: DAT SPECT is normal

##### Findings

Normal DAT uptake in the striatal regions.

##### Interpretation

A normal DAT SPECT would indicate that the dopaminergic system is relatively intact, which is more typical of AD than DLB. This finding would suggest that the patient’s symptoms are more likely due to AD or another form of dementia not associated with significant nigrostriatal degeneration.

##### Impact on management

This would support a diagnosis of AD over DLB, making the patient a suitable candidate for AD-specific treatments like monoclonal antibodies.

These two clinical scenarios, exemplified in Case 1 and Case 2, underscore the critical role of DAT SPECT imaging in the challenging differential diagnosis between DLB and AD. They resonate with findings from previous studies (25, 71) and, illustrating how DAT SPECT results can influence diagnosis and treatment strategies.

### 5.3 Case example 3: diagnosing MCI-DLB with sleep disturbances and medication implications

- A 68-year-old woman presents with a one-year history of mild cognitive changes. She complains of frequent vivid dreams and episodes of acting out her dreams during sleep, often resulting in injury to herself or her bed partner. Her husband reports that she talks, yells, flails her arms, and occasionally falls out of bed during these episodes. These episodes have been increasing in frequency over the past year. Additionally, she experiences constipation and occasional orthostatic dizziness. On cognitive testing, she demonstrates mild memory deficits, particularly in episodic memory, and difficulties with word recall. There are no significant Parkinsonian features on examination.

The patient’s presentation is suggestive of Mild Cognitive Impairment (MCI) with possible underlying Lewy body pathology, which is characterized by cognitive symptoms along with REM Sleep Behavior Disorder (RBD) and autonomic disturbances. Due to the complexity of her symptoms and potential differential diagnoses, further evaluation is warranted. Given the patient’s history of vivid dreams, acting out dreams during sleep, and cognitive deficits, a DAT SPECT is ordered to assist in the diagnosis. This case highlights the importance of DAT SPECT in patients with MCI-like symptoms where the presence of Lewy body pathology can be challenging to confirm based on clinical criteria alone.

#### Scenario 1: DAT SPECT reveals dopaminergic deficit

##### Findings

The DAT SPECT shows reduced DAT uptake in the basal ganglia, particularly in the putamen.

##### Interpretation

This result suggests the presence of dopaminergic deficits, which aligns with the presence of Lewy body pathology. The co-occurrence of RBD, cognitive symptoms, and autonomic issues points towards a diagnosis of MCI due to Dementia with Lewy Bodies (MCI-DLB).

##### Impact on management

This DAT SPECT result supports initiating treatment with a cholinesterase inhibitor, targeting the cognitive symptoms and potentially mitigating the sleep disturbances associated with DLB. Optimizing the dose of ChE inhibitors in DLB patients can improve cognitive, psychiatric symptoms and some aspects of REM Sleep Behavior Disorder. In addition to pharmacological treatment, educating the patient and her bed partner about RBD safety measures is essential to prevent injuries during dream-enacting behaviors. Such education should include strategies to secure the sleeping environment, possibly modifying the bedroom layout to reduce injury risk, and considering the use of bed rails or mattress on the floor if necessary.

#### Scenario 2: DAT SPECT is normal

##### Findings

The DAT SPECT reveals normal DAT uptake in the striatal regions.

##### Interpretation

A normal DAT SPECT suggests that dopaminergic deficits are not the primary cause of her symptoms. This finding raises the possibility of alternative diagnoses, such as pure autonomic failure (PAF) or non-neurodegenerative sleep disorders.

##### Impact on management

In the absence of dopaminergic deficits, a re-evaluation of the diagnosis is necessary. Medication management decisions may include discontinuing any medication primarily prescribed for tremors or psychosis if they were started empirically. Further evaluation by a sleep specialist may be required to address the RBD symptoms.

This case exemplifies how DAT SPECT can be instrumental in diagnosing complex cases involving sleep disturbances and autonomic issues and guiding medication management decisions to optimize patient care.

**Figure 2:**
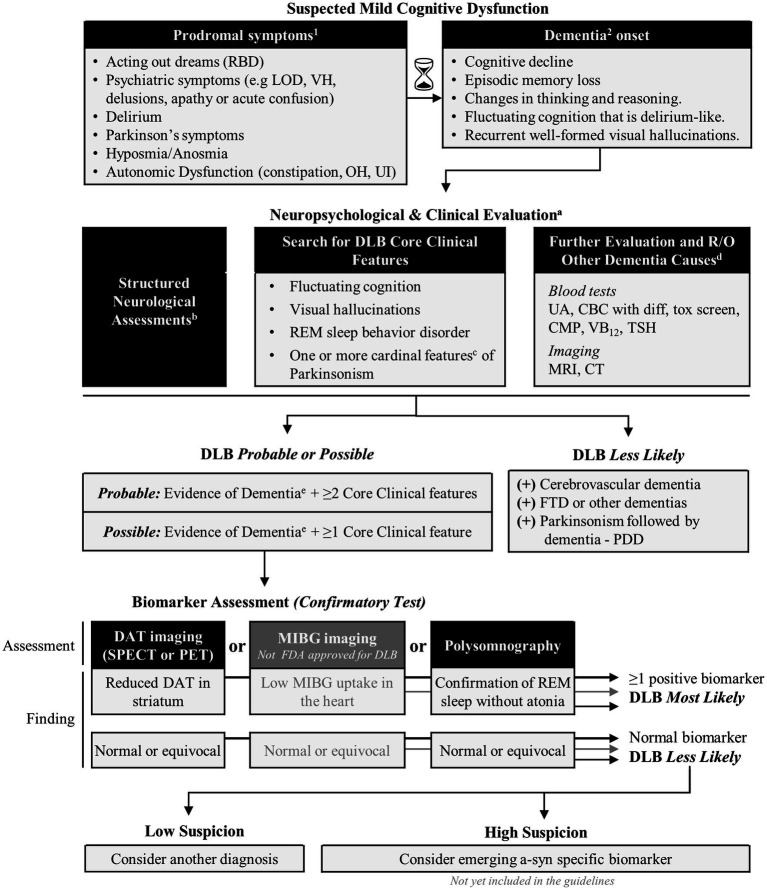
Proposed diagnostic algorithm for Dementia with Lewy Bodies (DLB).

To aid clinicians in determining the appropriate circumstances for the utilization of DAT SPECT imaging and/or other biomarkers in the diagnostic process, the “Proposed Diagnostic Algorithm for Dementia with Lewy Bodies (DLB)” is provided in Figure 2.

## 6 General considerations for the timing of DAT SPECT

Determining the optimal timing for ordering a DAT SPECT remains a pivotal question for clinicians. While the scan has evidence-based utility in differentiating between Parkinsonian syndromes, its exact role in the context of cognitive disorders, especially DLB, is nuanced. For instance, in patients presenting with cognitive decline that meets the criteria of dementia accompanied by subtle Parkinsonism or unexplained visual hallucinations, the DAT SPECT may offer a clearer diagnostic picture. However, in cases where the clinical presentation is unequivocally indicative of DLB (e.g., cognitive impairment plus two or more core features), the added value of using a biomarker may be more limited and should be weighed for risks, benefits, and implications for clinical management. In these scenarios, clinical judgment should take precedence.

Repetition of a DAT imaging is not typically a standard practice, especially in cases with entirely normal baseline SPECT or typical PD patterns (95). However, it may be considered in cases where there is an unexpected negative result in the face of strong clinical suspicion of a dopaminergic deficit disorder, when the visual read is indeterminant, or when the clinician’s suspicion of a suspected DLB remains strong over a period of time (74, 95). Again, clinical judgment should drive the decision to repeat imaging with appropriate discussions with the patient and family. Regarding the timing for repeat scans and changes over time, it is currently an area of ongoing research and remains relatively unknown; however, some studies, such as the Parkinson’s Progression Markers Initiative (PPMI) and others, provide valuable information on the rates of change in PD [for review see; (96–98)]. Generally, a repeat scan may be considered within 12–18 months for suspected DLB.

### 6.1 Limitations and challenges

It is essential to understand that a positive DAT SPECT, indicating dopaminergic deficits, does not provide a specific diagnosis but instead supports one. Furthermore, a negative result does not exclude diagnoses like DLB. Conducting a comprehensive review of DAT SPECT images in collaboration with a board-certified nuclear medicine radiologist, considering both visual rating and quantitative analysis, is recommended. This collaborative review should consider both visual rating and quantitative analysis, recognizing that DAT SPECT is a supportive biomarker that should be used in clinical symptoms and history. Notably, while DAT SPECT can help differentiate parkinsonian syndromes from other conditions, it does not distinguish among specific parkinsonian syndromes (such as PD, PSP, MSA, CBD, or DLB), and a holistic diagnostic approach should be employed to differentiate between them based on clinical features and additional diagnostic tests.

### 6.2 Technical factors

Clinicians, including neurologists, psychiatrists, and other healthcare providers, must consider several factors that can affect DAT SPECT appearances. Key factors include the radius of detector rotation, image processing, timing of injection, and patient movement during the scan. Additionally, variations in the guidelines or standards to interpret imaging can affect diagnostic conclusions and subsequent treatment decisions (65). Any patient movement during the scan can affect the scan’s appearance. Patients with dementia particularly may struggle to remain still during scanning, as scans take in the order of 20–30 min to perform and detectors rotate near the patient’s face. This can be a particular issue when interpreting scans for DLB, as appearances secondary to movement and scanning with wider radiuses of rotation can mimic those of more balanced loss. Proper training for radiologists and nuclear medicine specialists is essential to provide a trusted and accurate visual rating. Neurologists should also be comfortable with visual reads to provide clinical correlation and interpretation.

### 6.3 Impact of cerebral ischemia and structural lesions on DAT SPECT interpretation

Focal loss at the striata can be secondary to strategically occurring infarcts or lesions, making appropriate correlation with anatomical imaging imperative to reduce false positive results. Structural lesions, such as strokes, in the basal ganglia or along the course of nigrostriatal neurons can impact the interpretation of DAT SPECT results. These lesions may disrupt the basal ganglia’s typical anatomical structures and neural pathways, leading to changes in radiotracer uptake patterns. For example, a small midbrain lesion, a putaminal lesion or a large middle cerebral artery infarct can all give asymmetric striatal uptake patterns that can potentially mimic DAT SPECT findings seen in PD or DLB. Clinicians and nuclear medicine radiologists should be aware of the potential influence of such structural lesions on DAT SPECT images.

### 6.4 Radiation exposure considerations

It is essential to acknowledge that DAT imaging, like other medical imaging procedures involving radioisotopes, involves exposure to ionizing radiation (99). Clinicians and patients should be aware of the associated risks, particularly in cases where repeated scans are considered. The decision to undergo DAT SPECT should always be weighed against its potential benefits in aiding diagnosis and management. The Effective Dose resulting from a DaTscan administration with an activity of 185 MBq (5 mCi) is 3.94 mSv in an adult. Appropriate precautions, such as adhering to recommended safety protocols and frequent voiding, should be taken to minimize radiation exposure. Before consenting to the procedure, patients should also be informed of the risks and benefits.

### 6.5 Cost and accessibility

As a specialized imaging technique, DAT SPECT may not be readily available in all nuclear medicine imaging facilities in the US. Insurance coverage, cost considerations, and the availability of the scan in community settings can act as potential barriers and should be considered for discussions with patients/families.

It might be beneficial for practitioners to have identified resources or directories of local DAT SPECT imaging centers, along with experienced radiologists or nuclear medicine specialists, ensuring accurate visual reads and timely and efficient imaging for patients.

## 7 Considerations of DAT SPECT in prodromal DLB

A more recent addition to the literature is research criteria for prodromal states of DLB (14, 100), including mild cognitive impairment due to DLB (MCI-DLB). Similar in concept to MCI due to Alzheimer’s disease (MCI-AD), individuals with MCI-DLB have cognitive changes without sufficient interference in activities of daily living to meet the criteria for dementia (101). While core DLB symptoms are noted in MCI-DLB, they may be more subtle and less consistent. DAT SPECT may help discriminate MCI-DLB from MCI-AD in the proper clinical setting. A recent report suggests that most possible MCI-DLB and over half of probable MCI-DLB patients had a DAT SPECT within normal baseline limits, with progressive signal loss during follow-up (102). However, changes in DAT SPECT are more likely to occur in later stages of nigral degeneration. In two studies, baseline DAT SPECT imaging, rated visually, at the MCI-DLB stage had sensitivity from 54–66% and specificity from 88–89% (76, 100). DAT SPECT may help with clinical decision-making and enrich clinical trial recruitment, identifying MCI-DLB cases for enrollment, but further validation of these research criteria is needed before a full recommendation for use can be made.

## 8 Emerging biomarkers for DLB diagnosis

The development of plasma α-synuclein assays is in its early stages, yet promising methods like seed amplification assays have shown potential in detecting misfolded α-synuclein aggregates in CSF with high sensitivity and specificity (103–105). In addition, CSF exosomes from individuals with DLB have been found to contain pathogenic α-synuclein forms, indicating their potential as biomarkers (106).

Abnormal α-synuclein accumulation in peripheral tissues, especially cutaneous nerve fibers, has been identified as a sensitive indicator for DLB diagnosis (107). This α-synuclein deposition in peripheral regions seems more prominent in DLB than in other synucleinopathies (108). Recently, the development of biomarkers such as α-synuclein seed amplification assays and skin biopsies has been gaining momentum in the US (109, 110). However, these have not yet been incorporated into diagnostic criteria or fully validated in all clinical settings, and their current usage may be more suitable for research purposes only until their clinical validation has been completed. In the future, alpha synucleinopathies may be classified under the same umbrella (111).

Lastly, there is growing evidence of the potential of gastrointestinal biopsies, as demonstrated by a recent study that emphasized the concurrence between α-synuclein immunohistochemistry and PMCA analysis of α-synuclein aggregates (112). However, these methods are not yet FDA-approved for DLB, but they represent a promising avenue for future research and may complement the information obtained from DAT imaging. In sum, integrating emerging biomarkers for DLB diagnosis with DAT imaging can enhance the accuracy and specificity of DLB diagnosis in the future, especially in challenging cases.

## 9 Conclusion

DAT imaging is a valuable tool for diagnosing Lewy’s body disorders in some instances. ^123^I-ioflupane SPECT, a sensitive and specific modality for detecting dopaminergic degeneration, is now an FDA-approved tool distinguishing DLB from AD. Visual interpretation of the scans by experienced radiologists and nuclear medicine specialists provides radiological confirmation of dopaminergic deficits. In the presence of cognitive impairment, at least one core clinical feature (Parkinsonism, visual hallucination, cognitive fluctuation, RBD) meets consensus criteria for diagnosis of DLB. Beyond diagnosis, DAT SPECT’s value extends to informing treatment strategies and contributing to the development of new therapies through enriched clinical trial cohorts.

## 10 Summary: practical guidelines for neurologists regarding DAT scans

*Considerations for ordering DAT scan:*

- Before ordering a DAT scan, ensure the patient comprehends why it is recommended and the specific diagnostic challenges it addresses.
- Discuss the potential advantages of a DAT scan, such as its ability to distinguish between different movement disorders, while also highlighting its inherent limitations.
- Emphasize any possible interactions with medications or substances, ensuring the patient understands the importance of full disclosure regarding their substance use. Adjustments should be made accordingly.

*How to order DAT scan:*

Before the scan, delve into the patient’s complete medication and supplement list, highlighting agents that interfere with DAT imaging.

- If clinically safe and potentially beneficial, think about a brief cessation of certain medications that might introduce ambiguity into the results. Collaborate with prescribing physicians when considering this step.
- Recognize that substances, especially stimulants, can substantially alter DAT scan results. If possible and safe, advocate for a period of abstinence before the scan, ensuring the most accurate results.

*What to expect with the scan:*

- DAT SPECT has a binary outcome: normal/abnormal. Restrain from over-interpreting scans in clinical practice.
- Remember, a DAT scan is a supplementary tool; always juxtapose its results with the broader clinical picture, such as symptoms, history, and physical exam findings.
- Correlate the DAT scan findings with clinical symptoms to ensure diagnostic accuracy and avoid undue reliance on imaging.
- *False positives vs. False negatives:* acknowledge that while rare false negatives can occur (e.g., due to SSRIs/SNRIs), false positives due to drug interference or drugs of abuse are more prevalent. Approach interpretation judiciously, especially when scan results seem discordant with clinical presentation.
- For especially challenging scenarios, a multidisciplinary case conference can be invaluable.

*Interpretation and discussion with patient:*

- Dedicate a session post-scan to explain its results to the patient, ensuring they understand its implications and the next steps.
- Meticulously record the rationale for the DAT scan, its interpretation nuances, and all clinical decisions made post-scan to provide a comprehensive patient history.

*Accessibility and adherence:*

- Ensure this information is readily available for every healthcare professional involved in the patient’s care. Stress the significance of strict medication compliance and educate about potential side effects. The dynamic nature of many neurological conditions reinforces the need for regular check-ups and reassessments.

*Stay updated:* commit to continuous learning in the rapidly evolving realm of biomarkers. Regularly consult current literature, research, and guidelines to ensure top-tier patient care.

## Author contributions

DO’S: Conceptualization, Data curation, Formal analysis, Investigation, Methodology, Project administration, Writing – original draft, Writing – review & editing. AA: Conceptualization, Funding acquisition, Investigation, Resources, Visualization, Writing – original draft, Writing – review & editing. DG: Writing – original draft, Writing – review & editing. JGG: Writing – original draft, Writing – review & editing. ZS: Conceptualization, Writing – original draft, Writing – review & editing. GP: Writing – original draft, Writing – review & editing. JT: Writing – original draft, Writing – review & editing. JEG: Conceptualization, Methodology, Project administration, Resources, Supervision, Visualization, Writing – original draft, Writing – review & editing.
